# Plasma & Microwaves as Greener Options for Nanodiamond Purification: Insight Into Cytocompatibility

**DOI:** 10.3389/fbioe.2021.637587

**Published:** 2021-06-30

**Authors:** Dimitar P. Mitev, Amir M. Alsharabasy, Liam Morrison, Sebastian Wittig, Christof Diener, Abhay Pandit

**Affiliations:** ^1^CÚRAM, SFI Research Centre for Medical Devices, National University of Ireland Galway, Galway, Ireland; ^2^Earth and Ocean Sciences and Ryan Institute, School of Natural Sciences, National University of Ireland Galway, Galway, Ireland; ^3^Diener Electronic GmbH + Co. KG, Ebhausen, Germany

**Keywords:** nanodiamond, purification, modification, cytotoxicity, plasma treatment, microwave, nanocarbon, nanomaterial

## Abstract

The potential biomedical applications of nanodiamond have been considered over the last few decades. However, there is still uncertainty regarding the extent to which the surface characteristics of this material can influence potential applications. The present study investigated the effects of surface characteristics alongside the prospective of improving nanodiamond production using cold plasma and microwave technologies for the surface tailoring of the nanocarbons. Numerous approaches were applied to purify, refine and modify a group of nanosized diamonds at each step of their production cycle: from the detonation soot as the initial raw material to already certified samples. The degree of surface changes were deliberately performed slowly and kept at different non-diamond carbon presence stages, non-carbon elemental content, and amount converted superficial moieties. In total, 21 treatment procedures and 35 types of nanosize diamond products were investigated. In addition cultures of human fibroblast cells showed enhanced viability in the presence of many of the processed nanodiamonds, indicating the potential for dermal applications of these remarkable nanomaterials.

## Introduction

Micro- and nanosized drug delivery vehicles have certain advantages over standard drug delivery approaches in a range of cases. According to technical marketing research estimations, the global advanced drug delivery market is expected to grow steadily in the coming years, with compound annual growth rate between 5.3 and 7.0% (Fact. Mr, 2020; Mordor Intelligence, 2020). Nanodiamond, and particularly detonation nanodiamond (DND), is among the most reported nanoscale drug delivery systems to date (Castro and Kumar, 2013; Singhana et al., 2014; Zhang et al., 2014; Amenta and Aschberger, 2015; Jazayeri et al., 2016; Silva et al., 2016; Castro et al., 2017; Neburkova et al., 2017; Roy et al., 2018; Trofimov et al., 2018; Das and Huang, 2019; Ekladious et al., 2019). DND is a carbon derived nanomaterial that has become a promising candidate in biological and medical applications in recent decades. These include targeted drug delivery, biosensors, markers for cell imaging, and solid support for peptide synthesis. Moreover, these materials are used as structuring additives and bio-modifiers in composite biocompatible coatings for medical implants (Zhu et al., 2012; Kaur and Badea, 2013). A common problem for all the biological applications of DND is the limited availability of nanodiamond (ND) types with various surface chemistry. The desired properties can be gained through complex liquid-phase treatments, increasing the cost and thus limiting potential further use (Liu et al., 2004; Mitev et al., 2014a; Arnault, 2016; Turcheniuk and Mochalin, 2017).

There are many different routes for modifying nanoparticles used nowadays in biomedical applications (Sperling and Parak, 2010; Kango et al., 2013). The vast majority of these treatment and pre-treatment methods are liquid phase interactions. Some of them are hetero-phase involving gases or vapours and triggered by thermal/chemical vapour deposition (CVD) influence (Kalska-Szostko et al., 2015), ultraviolet (UV)-light, microwaves and, in some cases, plasma impact (Kim et al., 2013). The main advantages of cold plasma treatments are their output versatility (Morosoff, 1990), the possibility to outweigh a range of other methods, ability to extend the process to various degrees of completion for different targets and their economic performance on both small and large scales (Vossen and Kern, 1991; Diener Electronic, 2011; Schwander and Partes, 2011; De Geyter and Morent, 2012; Woodard et al., 2018).

Up to the present time, most of the publications about plasma interactions with NDs focus on the synthesis of these materials (Popov et al., 2004; Yafarov, 2006; Williams et al., 2008; Kumar et al., 2013; Gottlieb et al., 2016), with few dealing with plasma-assisted surface modification, and especially DND purification. Low-temperature plasmas of CH_4_/O_2_ mixtures were applied to change the presence of polar groups on ND surfaces (Yu and Kim, 2006). Attempts were made for hydrogenation and amination of DND by atmospheric pressure surface barrier discharge (Kromka et al., 2015; Jirásek et al., 2016a). The corona or spark discharges directly over suspensions targeted surface modification (Stenclova et al., 2017), or purification of raw detonation soot (DS; Medvedev et al., 2012). Reduction of surface sp^2^ type of carbon was also noted after applying pulsed corona discharge in saline solutions (Jirásek et al., 2016b), or directed RF plasma micro-jets (Kozak et al., 2016). A rare approach of “dry” plasma DND purification involved the employment of an atmospheric pressure oxygen plasma jet for digesting the surface of pre-formed DS pellets (Hong et al., 2017).

Related to the potential applications of DND as a drug carrier agent and its cytotoxicity, it is widely accepted that the role can be significantly influenced by the surface properties and the purity of this material. Considerable differences were found in the adsorption capability of DND against antibiotics, depending on the DND brand specifics, and the termination with COOH and NH_2_ groups (Mochalin et al., 2013). Roy et al. (2018) used a similar approach and termination to estimate the loading capacity of DND and cytotoxic effects in vitro. In contrast, no cytotoxic effects were found by Schrand et al. (2007) for carboxylated DND. Silbajoris et al. demonstrated that despite being non-cytotoxic to the human airway epithelial cells (HAEC) *in vitro*, the DND induced inflammatory gene expression in a dose-dependent manner (Silbajoris et al., 2009). Further research revealed that although the mechanism involved is dependent on the production of intracellular H_2_O_2_, suggesting that DND exposure presents oxidative stress to HAEC, further oxidation of the DND surface can reduce this effect (Silbajoris et al., 2015). The loss of non-diamond carbon from the DND surface correlated to a reduction in the inflammogenic activity, which was observed for the air-annealed samples following heating periods even as short as 5 min.

The main objective of this study was to assess how both carbon and non-carbon purity of DND impacts on its potential biomedical application. From an economic perspective, it is clear that the employment of greener alternatives for the purification and surface modification of this material is of paramount importance. These include avoiding the extensive use of chemicals during the liquid purification steps, skipping complex deaggregation during the surface modifications and getting even better results with simpler “dry” methods. This study will explore these alternatives and investigate how processes involved affect the cytocompatibility of the new NDs.

## Materials and Methods

### Sample Preparation

#### Samples for Plasma Treatments

Deionised (DI) water-based suspensions were initially prepared, containing 0.5 wt% of raw DS, or 0.4 wt% of already purified NDs (NSPA, MyD, or PlCh). After 30 min of ultrasonication (sonicator type B), microscopy glass slides (Carl Roth or Thermo Scientific SuperFrost) were placed on a horizontal surface in a fume hood and coated evenly with 1 mL of the corresponding suspension. Sample slides were ready for use after drying. This approach required only minute quantities of DND and thus was more flexible, than the rotation reactors. The treatment procedures were conducted according to Supplementary Informations 1, 2.

#### Samples for Microwave Digestions

##### Acid-dichromate Purification

About 2.019 g of DS was mixed with 10 mL of DI H_2_O, and 40.0 mL of concentrated H_2_SO_4_ which was slowly added using a magnetic stirring device, until complete homogenisation was achieved. Simultaneously with the addition of 23.5 g of K_2_Cr_2_O_7_ in portions, the temperature was increased and maintained within of the range of 90–110°C until the colour lightened from black to grey and gas emission was complete. This was followed by consecutive washings and sedimentations until reaching a pH 4 or higher. The yield after drying was 1.429 g (70.78%) of purified ND (type MyD).

##### Microwave-assisted DS Purification

Three different purification variants were applied (Supplementary Information 2B). Aliquots of 1.1 g DS were mixed with corresponding acid blends and agitated until fully suspended. Depending on the procedure, 5–5.5 mL of the suspension were transferred to each of the polytetrafluoroethylene (PTFE) digestion vessels and decomposed with an optimised microwave-induced digestion steps (MLS 1200 MEGA digestion system with 6-position rotor, Milestone, Sorisole, Italy). Following the treatment, the suspensions were washed repeatedly until pH 4 or higher (Mitev et al., 2014a).

##### Microwave-assisted DND Refinement

Five different complexant-driven refinement variants were studied, in comparison with the performance of the acid mixture of type NSPA (Mitev et al., 2014a; Supplementary Information 2B). Each treatment was performed for two different types of ND: acid-dichromate purified MyD and for a commercial one, denoted as PlCh.

### Characterisation

#### Techniques and Instruments

##### Infrared Spectra

The Fourier-transform infrared spectroscopy (FTIR) analysis was performed on Varian 660-IR FT-IR Spectrometer (Varian Australia Pty Ltd., Australia) fitted with Specac Quest ATR Accessory (Specac Ltd., United Kingdom). The scanning range was 600–4,000 cm^–1^ and the resolution was 4 cm^–1^. Total spectra with a background of air were collected for the ND, each as average of 30 scans.

##### Raman Analyses

All spectra were recorded using Renishaw inVia microRaman equipped with 514 nm laser, at 50% laser power (to avoid sample heating), and a Leica microscope with 50× objective lens.

##### SEM and EDX

For the S- and F-containing ND samples, corresponding SEM-EDX elemental analysis was performed using a Hitachi S-4700 SEM equipped with a Bruker X-Flash 6160 EDX detector. An accelerating voltage of 20 kV was used; all samples were Au-coated.

##### Inductively Coupled Plasma-Mass Spectrometry

Elemental determinations in the samples (32 elements) were performed using Inductively Coupled Plasma-Mass Spectrometry (ICP-MS; Elan DRC-e, Perkin-Elmer SCIEX, Waltham, MA, United States) in a class 1000 (ISO class 6) clean room.

##### X-ray Photoelectron Spectroscopy

X-ray photoelectron spectroscopy (XPS) was carried out in a Kratos Axis ULTRA Spectrometer (Kratos Analytical Ltd., United Kingdom) using monochromatic Al Kα radiation of energy 1486.58 eV. The XPS detection limit was ∼0.1 at%; analysis area approximately 1 mm^2^ and the depth of analysis ∼10 nm. C1s line at 284.8 eV was used as charge reference. Elemental analyses were obtained from a survey spectrum of the entire binding energy; high-resolution spectra were also taken at a number of photoelectron transitions (pass energies correspondingly: 160 vs. 20-eV, steps 1.0 vs. 0.05 eV). Construction and peak fitting of synthetic peaks in narrow region spectra used a Shirely type background and the synthetic peaks were of a mixed Gaussian-Lorenzian type.

##### Contact Angle Measurements

Contact angle measurements were performed with an OCA 15EC Instrument fitted with single direct dosing system SD-DM and SCA 20 contact angle software (DataPhysics Instruments GmbH, Germany). One microlitre drops of D water were deposited on the surfaces at room temperature, and analysed by the sessile drop method. Thirteen microlitre drops were used for superhydrophobic layers.

##### Profilometry

A Veeco DekTak 150 stylus profilometer was utilised for determining the film thickness of plasma-polymerised allyl amine films.

##### Zeta potential/size

Zetasizer Nano-ZS90 particle analyser (model ZEN3690; Malvern Instruments Ltd., United Kingdom) fitted with 4 mW 633 nm (red) He-Ne laser was used. The electrokinetic or zeta potential (ZP) was derived from the electrophoretic light scattering of the suspended particles, at 25°C in DI. Reported values are averages of three measurements (each consisting of an average of 10–15 runs).

##### pH

SevenEasy pH (Mettler-Toledo, Switzerland). Suspensions mixed with the probe to equilibration, then probe left static without contact with the walls to provide consecutive steady reading.

##### BET

Low temperature nitrogen adsorption on Autosorb 1 instrument (Quantachrome Instruments, United States); the NSPA ND sample was 29.1 mg, vacuum heated for 14 h at 100°C, with SSA 259.43 m^2^ g^–1^, pore volume 1.372 cm^3^ g^–1^ and pore radius of 80.541 Å.

##### Colourimetry

HP Scanjet 7400c flatbed scanner, with powders deposited onto the glass panel and covered by white paper sheet. The colorimetric estimation was based on black/white ratio, %, for each particular sample. Colorimetric measurement of the darkness of scanned images for purified and dried DND samples was done using Adobe Photoshop CS6 in grayscale mode; sampling from random squares 101 × 101 pixels.

##### Sonication

Ultrasonic baths of following types were used: (A) Elmasonic S 30 H (Elma Schmidbauer GmbH, Germany); 280 W, 37 kHz and (B) Sonomatic model S1800 (Langford Ultrasonics, now EJ Electronics Limited, United Kingdom); 450 W, 33 kHz.

##### Statistical Analysis

Two-way ANOVA was used with Holm-Sidak’s post hoc test, as more powerful than the tests of Tukey and Bonferroni. The comparisons were done by factor ‘Treatment’ between the groups (^∗∗∗^*p* < 0.001; ^∗∗^*p* < 0.01; ^∗^*p* < 0.05; ± SD).

#### Cell Viability

Alamar Blue (AB) assay was performed to evaluate the metabolic activity of human dermal fibroblasts (HDFs) in contact with the nanoparticles. The ND samples were first heated at 130°C for 30 min, then cooled down. After weighing, the nanoparticles were sterilised by UV irradiation for 30 min before suspending in heat-inactivated FBS, followed by sonication for 45 min at room temperature. With continuous vortexing, four different concentrations of nanoparticle suspensions were prepared in DMEM (Dulbecco’s modified Eagle’s medium; Invitrogen) containing 10% (v/v) FBS (Fetal bovine serum, Sigma) and 1% penicillin/streptomycin. The cells were harvested using 0.25% of trypsin-EDTA and seeded at a density of 15 × 10^3^ cell/well, where the cell suspension was mixed with the nanoparticle suspension and maintained in a humidified atmosphere of 5% CO_2_ and 20% O_2_ for 3 days. The final tested concentrations of the nanoparticle suspensions were 10, 20, 40, and 80 μg mL^–1^. For testing the metabolic activity, the culture media with any suspended nanoparticles was discarded, and each well was washed by PBS, followed by incubation of the cells with 200 μL fresh Cell-Quant^TM^ AlamarBlue Cell Viability Reagent (10%) for 4 h at 37°C in 5% CO_2_. The solution’s fluorescence was measured at excitation 550 nm and emission 590 nm on a VarioskanFlash-4.00.53 microplate reader (Thermo Fisher Scientific Oy, Finland). After discarding the AlamarBlue reagent, each well was washed with PBS and the cell proliferation was quantified by Quant-iT^TM^ PicoGreen^TM^ dsDNA Assay kit according to the producer’s protocol.

#### Variants for Nanoparticle Deposition

Dried suspension (DriedSUSP): suspension of 200 μg g^–1^ ND type NSPA was prepared in 70 vol% ethanol/water by bath ultrasonication for 10 min. Aliquots of the suspension were dispensed into the wells to match the desired concentrations (10–20–40–80 μg mL^–1^) and left to dry in a fume hood. Non-centrifuged suspension (NonCENTR): suspension of 2 mg mL^–1^ ND type NSPA was prepared in heat-inactivated FBS by bath “in-focus” ultrasonication for a period of 10 min, and aliquots of it were mixed in situ with DMEM and HDFs cell suspension and maintained as outlined above. Centrifuged suspension (CENTR) was prepared as previously described, with an additional centrifugation stage (whole well plate for 3 min at 4,000 rpm), before the addition of DMEM and HDFs.

## Results and Discussion

### Microwave-Assisted Treatments

Microwave processing has proved its efficiency in purifying NDs from non-diamond carbon and metals, plus other hetero-admixtures in the detonation soot—the initial raw product of the detonation synthesis of this material (Greiner et al., 1988; Mitev et al., 2014a). Further complexants-driven purification of DND is also possible: the process that we denote as refinement here.

The logical steps here, and for all other treatments, followed the sequence variation—characterisation—cytocompatibility.

#### Microwave Purification

**FIGURE 1:**
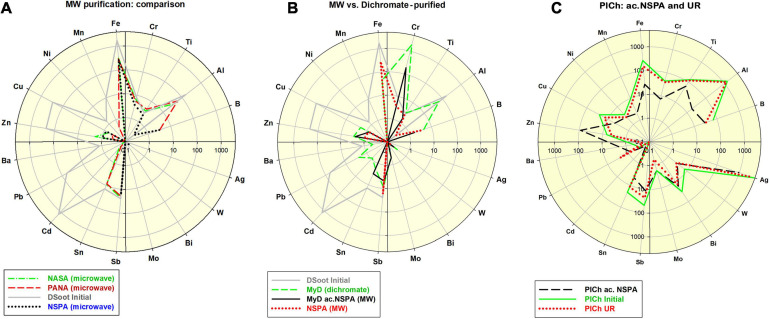
**(A)** Comparative distribution of 18 non-carbon impurity elements in the initial DSoot, vs. microwave-purified DND of types NASA, PANA and NSPA (ppm, logarithmic scale 0.1–4,000; check also Supp. Information 4); **(B)** Elemental distribution of acid-dichromate purified DND type MyD, compared with that of the parent DS, MW-purified NSPA and MW-refined MyD (MyD ac.NSPA); scale 0.1–5,000; **(C)** Elemental content of initial PlCh, PlCh ac.NSPA and PlCh UR; scale 0.1–5,000.

In order to further investigate the aspects mentioned above, one type of raw unpurified DS with an elevated hetero-elemental content of 4,034 ppm was studied (section “Microwave-Assisted DS Purification” and Supplementary Informations 1–3). The application of the procedures under protocols NASA, PANA, and NSPA was capable of diminishing nearly 13-fold this level, to correspondingly 467, 403, and 311 ppm. The most significant reductions were observed for Fe, Cu, Zn, and Cd, while Al was reduced up to three times by NASA and PANA, and entirely by NSPA. Only Sb remained virtually unaffected (Figure 1A). All analyses were based on direct ICP-MS of 0.01% aqueous suspensions of corresponding DND, according to the procedures in (Mitev et al., 2013).

#### Microwave Refinement (Acids)

Using benchmark samples of DND was essential for accurately estimating the final output after the MW procedures. For this purpose, and following an acid-dichromate purification procedure for the initial DS, DND rich in surface metal contaminants was produced. This sample, here denoted “MyD,” was abundantly enriched in Cr (8–1929 ppm), with other elemental content reduced though. The impurity level generally dropped twice from 4,034 ppm (DS) to 2,122 ppm (section “Acid-dichromate purification” and Figure 1B).

Together with MyD, a commercially available DND brand was used for the same purpose: PlasmaChem’s PL-D-G. This sample denoted here as “PlCh,” had a total impurity level of 6,700 ppm with elevated concentrations of Al, Fe, Ti, B, Sb, Cr, Cu, and enriched in Ag [4,588 ppm, as previously found for this brand (Mitev et al., 2014b)].

Both of these benchmark samples were subjected to a refinement procedure under the protocol NSPA, the same as the one used to purify DS. The Cr-content of the resulting ND type of “MyD ac.NSPA” diminished tenfold, and with a total of 254 ppm, the impurities dropped 16 times vs. the initial DS and 8.4 times vs. MyD. Direct digestion of DS under the NSPA protocol resulted in DND with only slightly higher levels of impurities (Figure 1B). An application of the same procedure in the case of commercial PlCh led to a 5.4 times drop for the total impurities to 1,252 ppm, mainly due to the reduction of Ag and the almost total elimination of Al and Fe (Figure 1C).

#### Microwave Refinement (Complexants)

**FIGURE 2:**
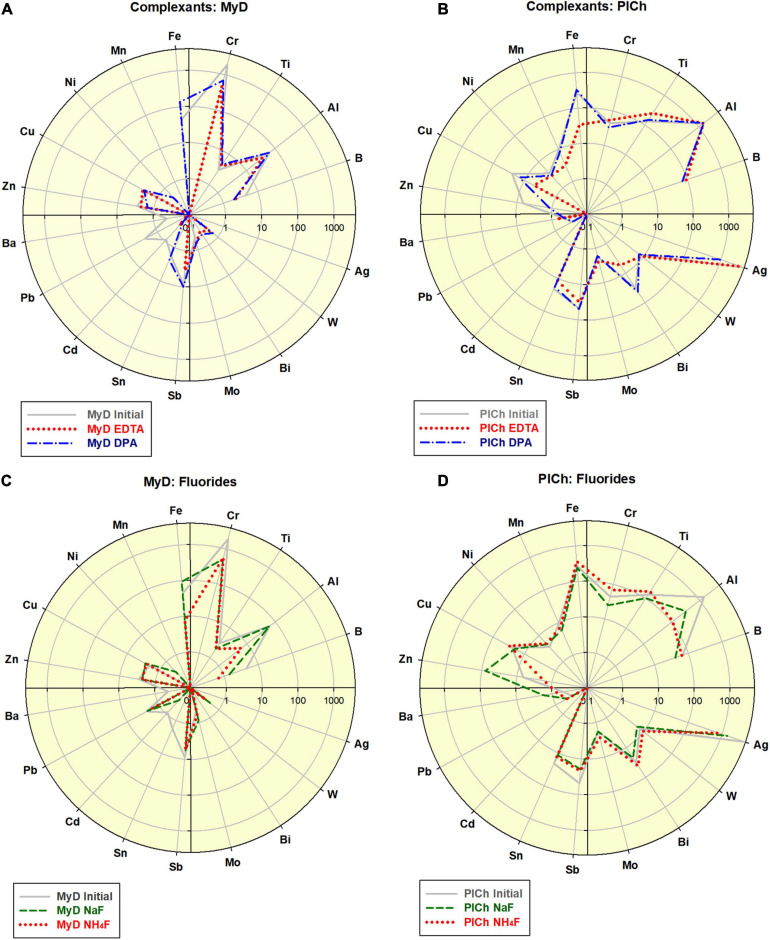
Effects of the complexant-driven refinement (Na_2_EDTA and 2,6-Pyridinedicarboxylic acid (here indexed “EDTA” and “DPA”; see also Supplementary Table B2): **(A)** treatment of MyD; scale 0.1–4,000; **(B)** treatment of PlCh; scale 0.1–5,000. Effects of the fluorides-driven refinement (NaF and NH_4_F; see also Supplementary Table B2): **(C)** treatment of MyD; scale 0.1–4,000; **(D)** treatment of PlCh; scale 0.1–5,000.

As a “mild” option of the MW assisted refinement, a range of complex-forming agents and inorganic fluorides were also used instead of the aggressive and toxic acid mixtures, such as those containing HF. Both of the benchmark DND samples underwent elemental impurity reduction, as outlined in Figure 2 and previous Figure 1C.

In the case of MyD, the total impurities were reduced by 2.3-fold using DPA and 4-fold using EDTA, which is comparable to the acid-driven treatments. The high Cr-content was reduced 2.8 times by DPA, and even a more effectively 4.1 times by EDTA. For PlCh, the latter complexant had a more substantial effect on Fe, with less effects on the high Ag levels. In contrast, DPA diminished Ag nearly 5-fold and, and for this reason primarily, the total impurity content dropped from 6,700 to 3,268 ppm (PlCh DPA; 5,905 ppm for PlCh EDTA). The performance of hexamethylenetetramine (Figure 1C) was very similar to that of EDTA, but with greater efficiency for the removal of both Fe and Al.

Apart from the metal content, ATR-FTIR spectra of ND surfaces did not reveal any significant change in the chemical composition after the refinement procedures. Most characteristic for all samples are the peaks around 1,100 cm^–1^ (C–O and C–O–C moieties), 1,260 (C–O stretch, N-defect, C–N stretch), 1,330 (–C–H bending, C–C stretch), 1,641 cm^–1^ (bending of O–H) and 1,730 (C=O stretch) (Petit and Puskar, 2018; Shenderova O. et al., 2011; University of Puget Sound, 2019).

### Plasma-Assisted Treatments

Plasma purification of raw DS and plasma modification of the already purified DND were included in the current study. In all cases, dry material was deposited on glass slides, with or without use of a Faraday cage, for the purification process.

#### Plasma Purification

**FIGURE 3:**
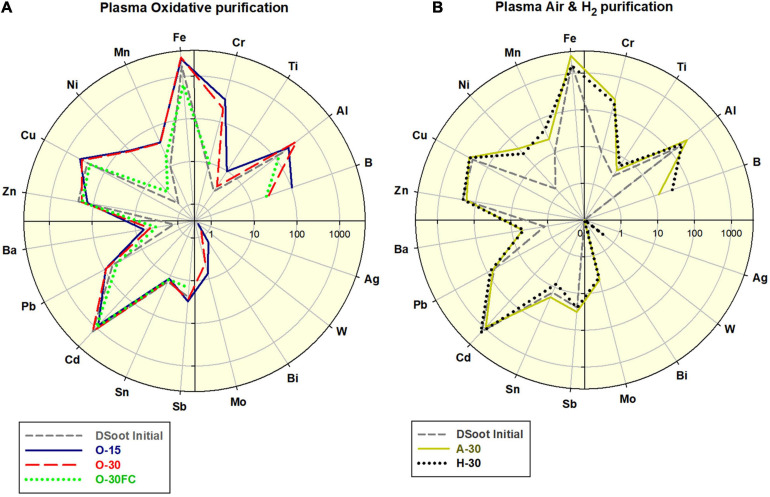
**(A)** Comparative elemental distribution of O_2_-plasma treated DS; **(B)** Air- and H_2_-plasma treated DS; scales 0.1–4,000.

All of the PL-purifications conducted were effective in the digestion of non-diamond carbon from the initial DSoot. As a single step, the plasma-assisted purifications can not reduce the levels of the elemental contaminants in the DS, where only the non-diamond carbon, organic and volatile admixtures can be oxidised and eliminated (Figure 3). The observed moderate increase of certain metals (Fe, Cr, Ni, and Mn) corresponds to the stainless steel content of the metal plates used. The difference between the FC-plasma-treated and the directly plasma-treated samples was significant. For the latter a 4.39 fold increase in Fe was observed along with apparent increases for Cr, Mn, Ni, and Cu. With the protective role of a Faraday cage, the elemental pattern for O-30FC was very similar to that of DS unlike all patterns of the direct-plasma-oxidised DS.

**FIGURE 4:**
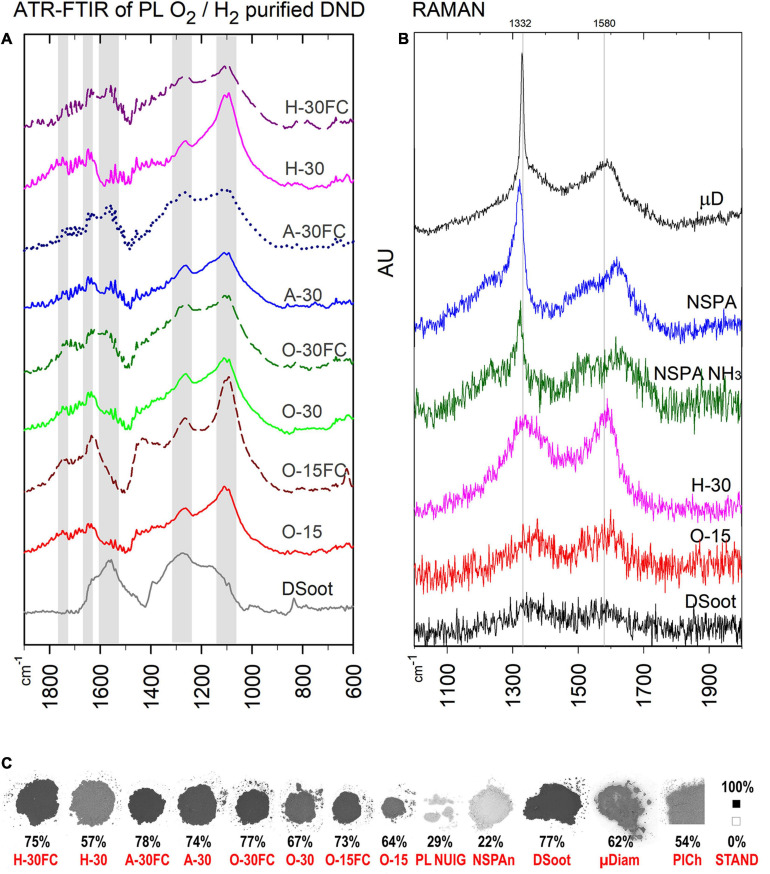
ATR-FTIR **(A)** and Raman spectra of PL- and MW-treated DND **(B)**, as compared with the parent DS and HPHT micronsized diamond as a standard (Raman; the top). **(C)** B & W colour ratios for all plasma-purified DND, parent DS and several others (bottom; the percentage shows the relative darkness of the powder; far right: B & W standards, the black square is with the used 101 × 101 pixels sampling size).

Visually, the procedures caused definite lightening of the directly treated soot, with a moderate change of the samples under FC-conditions (Figure 4C and Supplementary Information 7). Direct colorimetric assessment of the output powders gives a range of 57–78% of the black-to-white colour ratio for the PL-purified samples (Tetra instrument), 22% for the MW-purified NSPA, with the background of 77% for the initial DS. The lightest sample amongst the PL-treated was PL NUIG, and this effective removal of darker non-diamond carbon was achieved with the small Zepto system. At first sight it may look as a paradox for its power of 30W. Still, considering the small volume of the working chamber, identical frequency and treatment time, it is obvious that the plasma power density was highest here, and hence the effect.

Estimating the changes in the sp^3^/sp^2^ ratio of available carbon and the CNO elemental interrelation are indispensable tools in revealing other aspect of ND’s purity. XPS clearly shows significant enrichment in the sp^3^ diamond content, with some exciting peculiarities, however (Supplementary Information 4). Despite its darker appearance, O-30FC exhibited a higher level of sp^3^-C than the directly plasma-oxidised O-30 (78.3 vs. 67.6%), making it very similar in its properties to the cleanest MW-purified NSPA. Furthermore, the hydrogen plasma purification and gasification of the non-diamond carbon resulted in elevated sp^3^-C up to 76.9%, making this procedure completely competitive with the oxidative ones. Despite looking unusual here, the highest oxygen content is a result of the post-processing reaction of the diamond surface radical sites with the atmospheric oxygen (Khelifa et al., 2016). Amongst the elemental levels, the stable amount of nitrogen was also noteworthy. The fact that, regardless of the plasma treatment, it constantly fluctuated around 1.7–1.8 at% (besides the aminated DND), suggested that at least a significant portion of it is lattice-included (Shenderova O. A. et al., 2011).

The ATR-FTIR spectra of the plasma-purified DS direct us to the same conclusions (Figure 4A). Two features stand out most here: (1) for lighter direct-plasma treated samples—the disappearance of the pronounced peak at 1,566 cm^–1^, attributed to the sp^2^-coordinated carbon and characteristic for DS, was still pronounced for the darker “FC”-samples. (2) The peak around 1,100 cm^–1^ (C–O and C–O–C as outlined previously) is strong for all samples after plasma treatment and confirms the formation of an oxidised surface.

The Raman spectra correlate with the black and white colour ratio (Figure 4B): the lighter the sample, the more pronounced is the first-order Raman signal of diamond, a sharp asymmetric peak centred at ∼1,332 cm^–1^. The broad G-band in the 1,400–1,800 cm^–1^ range is associated mostly with non-diamond sp^2^ carbon, sp^2^ clusters and mixed sp^2^/sp^3^ carbon forms (Mermoux et al., 2018).

#### Plasma Modifications

A range of plasma-assisted surface modifications was performed to additionally extend the gamut of NDs available for cells’ response/cytotoxicity surveys. The modifications generated aminated, allyl-aminated, sulfhydrylated and fluorinated samples (Supplementary Information 2). The derivatives of NSPA type of DND are recapitulated below.

##### Elemental Changes

The increase in nitrogen levels was well expressed for the NSPA-NH_3_ (XPS, Supplementary Information 4), which raised from 1.7 to 1.8 at N%, to nearly 2.50 at% N in the aminated DND.

**FIGURE 5:**
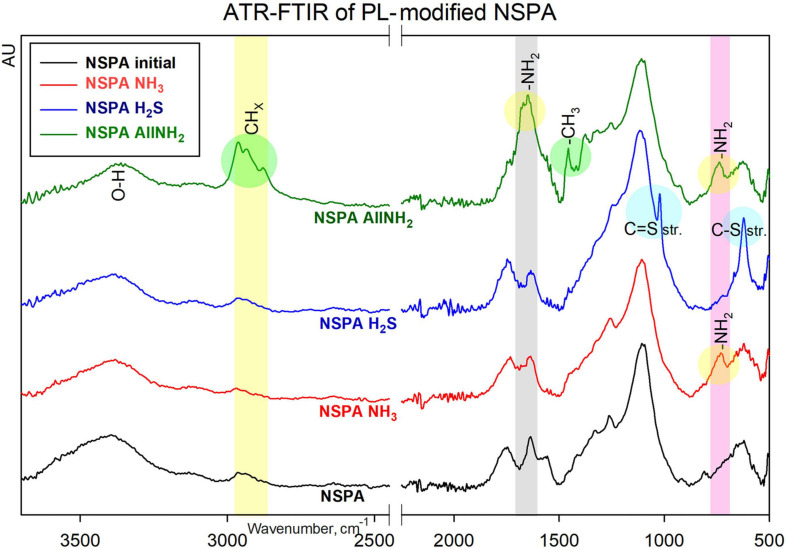
ATR-FTIR spectra of PL-modified NSPA type of DND.

The FTIR spectra also confirm an increase of the surface amine moieties (Figure 5), where the same characteristic absorbance at 730–738 cm^–1^ (N–H; out-of-plane bending) (Chemistry, 2012) appeared for both aminated NSPA NH_3_ and allyl-aminated NSPA AllNH_2_. For the latter, the strong peak at 1,647 cm^–1^ is associated with the in-plane bending of primary amines (Chemistry, 2012), where the NH_2_-groups are on the hydrocarbon chains. This was accompanied by a simultaneous rise at 2,880–2,960 cm^–1^ for CH_*x*_ and 1,457 cm^–1^ for the CH_2_-bending vibrations. The successful sulphur attachment was also pronounced, where peaks at 1,021 cm^–1^ (C=S stretch) and 622 cm^–1^ (C–S stretch) were observed (Hampton et al., 2010).

**FIGURE 6:**
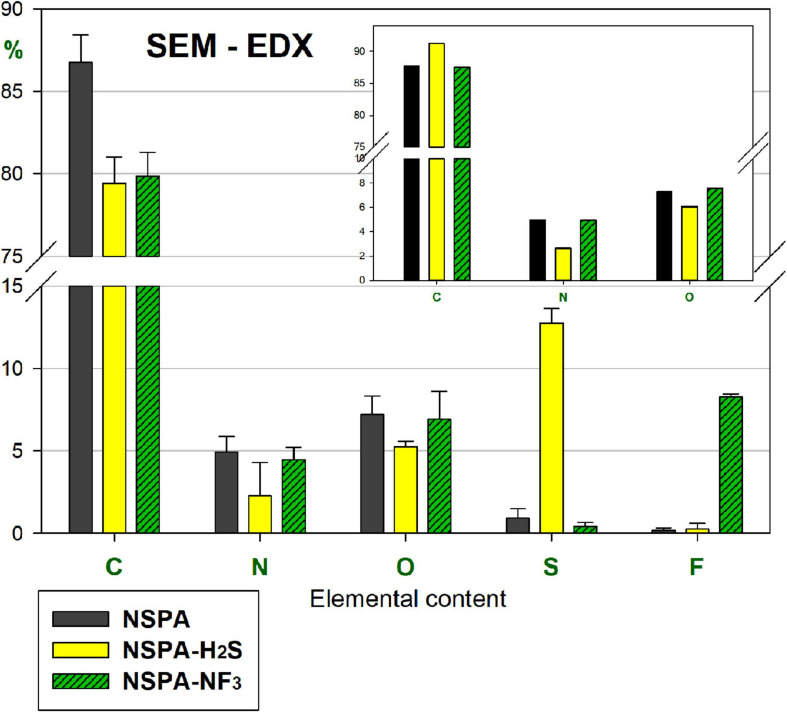
SEM-EDX elemental content of PL-sulphhydrylated and fluorinated samples, as compared with parent NSPA (“CNO-only” comparison: the inset plot at top right).

The SEM-EDX survey (Figure 6) revealed a ∼14-fold sulphur content jump from 0.93 to 12.76%. Furthermore, the “CNO-only” elemental comparison confirms these results (inner plot top right in Figure 6), where the sulphhydrylation changed that ratio compared to the initial NSPA, with a reduction in O and N, but an increase in C. This partial elimination of the first two elements proves the attachment of sulfhydryles directly to the surface and indirectly through the transformation of the parent moieties. In contrast, fluorine attached directly to the surface, without any change in the initial CNO-ratio.

##### Surface Effects

The plasma modification induced changes in the hydrophilicity of the thin layers. The contact angle (CA) of water drops, deposited on nanomaterial-coated glass slides showed distinct differentiation. Untreated DS and NDs of all three brands absorbed the drops completely in 3 s after deposition. The same behaviour was observed for the aminated and sulfhydrylated ND layers (Supplementary Information 5, row 1). The allyl-amination raised the hydrophobicity to 28.2 ± 0.7°. In comparison, the CA of a plain slide, plasma-treated in parallel, equals 59.7 ± 0.9° (row 2, AllNH_2_, coating thickness 45.7 nm).

The most drastic change was observed after the fluorination processes. For instance, both DS and NDs became so hydrophobic that a 1 μL DI water drop cannot detach from the capillary tip. Upon repeated injections, the drop detached only after reaching a volume of 13 μL (shown CA 145.1°). These layers were extremely non-adherent and sensitive to static electricity.

##### Electrokinetic Potentials

**FIGURE 7:**
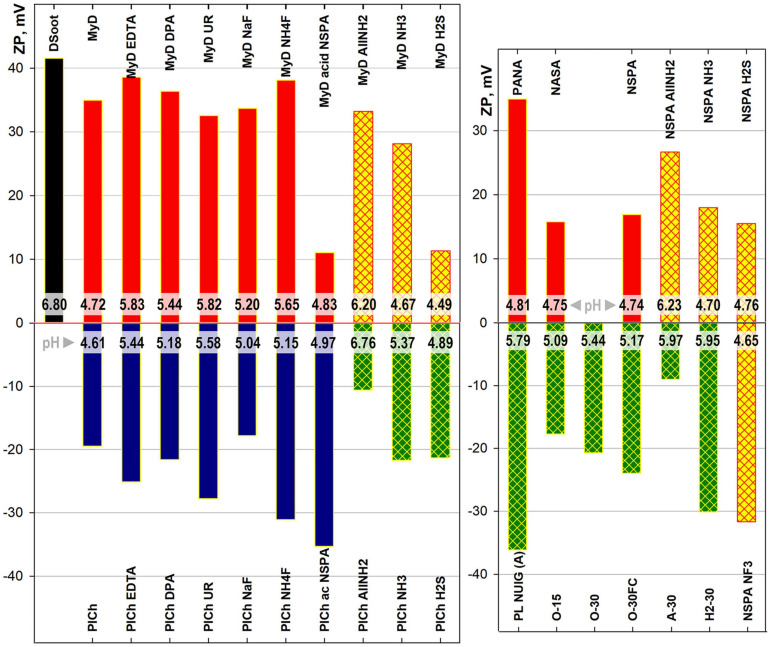
Electrokinetic (zeta-) potential and intrinsic pH ranges (strips close to base) of 0.05% ND suspensions in DI water.

Comparing the electrokinetic shifts of MW-treated, plasma-modified DND and the parent samples revealed the multi-faceted nature of zeta potential origin in complex colloids such as detonation NDs (Figure 7). In addition to their obvious importance in tracking DND suspension behaviour and deposition interactions, the zeta potentials are closely related to the non-carbon admixtures, adsorbed ions, and the sp^2^-C presence. They can be used as additional markers of the treatment efficiency. Several of the most pronounced tendencies were highlighted here:

A) *Microwave-assisted treatment:* Neither purification nor refinement changed the sign of the derivative samples, related to that of the parent sample. The two major DND types used throughout this study had opposite surface charges, positive for DSoot/MyD-derived samples and negative for PlCh and related. Despite being identical in chemical terms, the processes resulted in only relative shifts on the same side. This relates, on the one hand, to the persistent difference in the oxygen content following the samples’ detonation synthesis type and purification (most significant here). While for the PlasmaChem’s PL-D-G01 (dry detonation synthesis, CO_2_ cooling, HNO_3_ purification), the total oxygen content by XPS was equal to 16.5 at% (Mitev et al., 2014b; Garriga et al., 2020), similar type of NSPA purification for DSoot (wet detonation synthesis, H_2_O cooling) elevated the oxygen content from 3.86 to 7.24 at% (XPS, Supplementary Information 4). On the other hand, the adsorption of potential-determining ions onto the ND surface, especially when rich in polyvalent oxides, also plays a secondary role (Atkinson et al., 1967; Samuel de Lint et al., 2003; Peristyy et al., 2014; Vereda et al., 2015). The acid-dichromate purified sample MyD, abundant in Cr (ICP-MS, Supplementary Information 3), had a strong positive zeta potential (ZP) value of +34.9 mV. An application of the same type of NSPA purification procedure in parallel to the parent DSoot (sample NSPA) and the metal-rich MyD samples (sample MyD acid NSPA) led to a significant difference in zeta potentials, based only on the type of the metal present—Cr or Fe.
B) *Plasma-assisted modifications:* These maintained the above tendency, with only one exception—fluorinated NSPA NF_3_. Here, the high fluorine content and the direct chemical bonding to the carbon surface [displacing H (Nesvizhevsky et al., 2018)] rendered this surface very similar to that of highly fluorinated polymers. The ZP value of −31.7 mV at pH 4.65 is nearly identical to that of a PTFE suspension (Zhang et al., 2017), and higher than that of PVDF (Anton Paar, 2019). Similar to the mentioned fluoropolymers, the strong negative ZP is based on the preferential adsorption of hydroxide ions on an inert non-ionic ND surface (Beattie, 2006). These findings together with the observed strong increase in fluorine content (Figure 6) and the high hydrophobicity of NSPA NF_3_ (Supplementary Information 5; Barišić et al., 2021) prove the outright success of this plasma modification.
C) *Plasma-assisted purifications:* These dry processes change the situation completely. All the oxidative plasma purification/digestion processes convert the highly positive DSoot (+41.5 mV) to negatively charged samples. The degree of electronegativity correlates significantly with the oxygen content and the black and white colour ratio, with some particularities, however. These short 15–30 min procedures elevate the bound oxygen nearly threefold (Supplementary Information 4), thus overcoming the other mentioned factors and switching the ZP sign. Moreover, the relative weight of the sp^2^-C level can be employed as a “positive” ZP determinant (Gines et al., 2017). Even though the Faraday-cage-treated O-30FC showed a lower O-content of 9.48 at%, it exhibited more negative ZP than the direct plasma-treated O-30 (10.17 at% oxygen; ZP −24.0 vs. −20.7 mV). The higher sp^2^-C of O-30, caused by direct ion bombardment in the plasma chamber, outweighs the effect of oxygen increase and reduces the ZP negativity.

By comparing these results with previous findings (Mitev et al., 2014a), it becomes obvious that the oxygen moieties are not the only determining factor when the oxidative treatments are concerned. The ZP for DND is a combination of: the oxidised diamond surface, other heteroatom moieties, non-diamond carbon and (chemi)sorbed ions and compounds (Desai and Mitra, 2013; Peristyy et al., 2017; Shenderova and Nunn, 2017; Aizawa, 2019), with variable “relative weight” in each case.

### Cytotoxicity and Cell-Related Effects

As a final assessment for the interaction between cells and the carbon nanomaterials’ cells, the cytotoxicity of all initial, MW- and PL-treated samples were tested in vitro against human dermal fibroblasts (HDFs). Looking at the prospective dermal applications of potential ND drug carriers, the HDFs were chosen as the most appropriate for the test. The cells were cultured in the presence of nanocarbon samples over the total duration of the experiments. Here the target of complete deaggregation of ND was not pursued, considering the initial dry state of nanocarrier layers during the storage periods of hypothetic dermal products. Moreover, for the purpose of comparison, deaggregated commercial products were included and their effects on cell viability were evaluated.

We must highlight here the specifics of our procedure. Precipitated ND particles were still existent during the incubation with the AlamarBlue reagent, after the indicated culture period with cells. Moreover, the attempts to validate the metabolic activity results with the consecutive PicoGreen (PG) cell proliferation assay results, under these conditions were not successful to a great extent (Supplementary Information 6). This, according to our point of view, was connected with the different responses of these cell-based assays to small molecules and to nanomaterials. For instance, the quenching effect of divalent metal ions on PG fluorescence was described to be as high as 74% (Heaton, 1999). Here we highlight the metal range determined by ICP-MS for DND, and supported by the previous findings (Mitev et al., 2013, 2014b). Serious enhancement or the opposite, quenching of the PG fluorescence was caused by the presence of some metal nanoparticles (Dragan et al., 2010; Zinchenko et al., 2014), while an increase accompanied the crowding of negatively charged nanoparticles (Pustovit and Shahbazyan, 2012). In addition, the quenching effects of carbon nanotubes were also revealed to be a consequence of PG dye exclusion via the prevention of dye intercalation, due to DNA condensation around the nanotubes (Singh et al., 2005).

**FIGURE 8:**
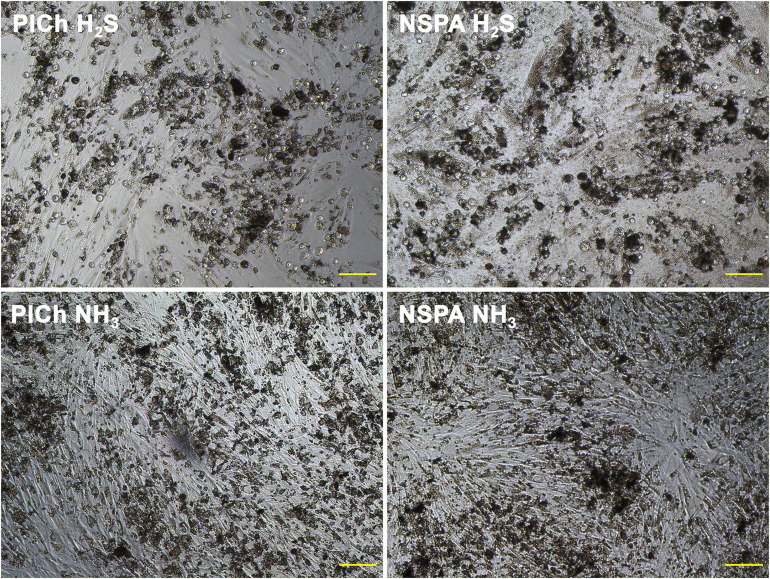
Cell cultures of HDFs in presence of cytotoxic “H_2_S-treated” ND (above) vs. beneficial plasma-aminated ND of the same types (below). Scale bar: 100 μm.

In the current study, the agitation throughout the freeze/thaw lysis procedures involved further adsorption of DNA and the fluorescent dye onto the ND aggregates, known to have a significant adsorption efficiency (Hemelaar et al., 2017). Depending on the degree of ND’s deaggregation during these procedures, or their surface characteristics, a significant enhancement or suppression of the fluorescence occurs. Such interference was not significant for the primary long incubation (4 h) with AB, as the sediment of ND was not mixed or agitated within the wells. Moreover, the measurement of the fluorescence of the reagent occurred separately from the cells and ND. Hence, due to the observed deviations, optical microscopy images were additionally taken as a verification procedure during all stages of the viability trials and used in a complementary manner (Figure 8).

#### Influence of the Nanoparticle Deposition on the Experimental Output

**FIGURE 9:**
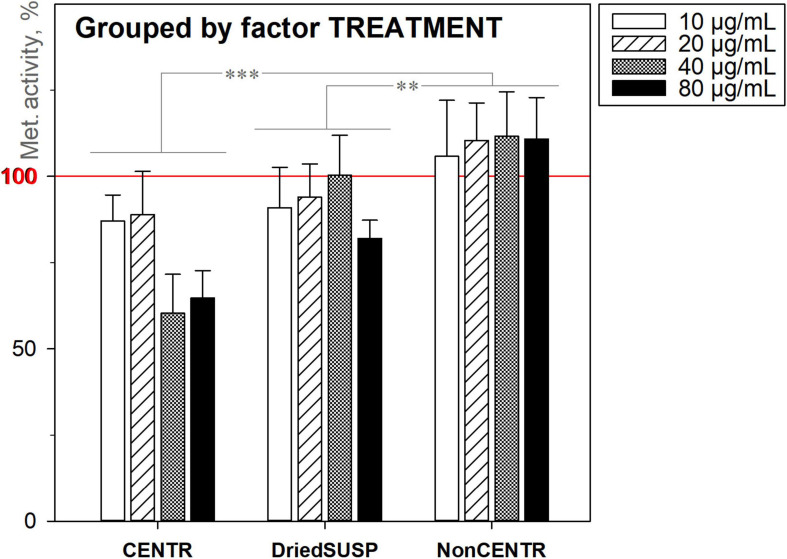
Metabolic activity of HDFs cultured with DND for 72 h under three deposition methods, using the alamarBlue^TM^ assay (normalised to the non-treated cells; *n* = 4; ^∗∗∗^*p* < 0.001; ^∗∗^*p* < 0.01; ^∗^*p* < 0.05; ± SD).

A parallel comparison of three different ND deposition methods (Figure 9; section “Characterisation”) clearly showed that despite using identical amounts of DND for each group (10, 20, 40, and 80 μg mL^–1^, 200 μL per well), ultimately the deposition specifics is of crucial importance. This can relate to: (a) the sorption capacity of the particles, where the ND adsorbs various nutrients from the culture media, and delivers them in a “concentrated” form directly to the cells and (b) bigger nanoparticle aggregates and agglomerates provide additional surfaces for the cell attachment and proliferation (the preliminary centrifugation or deposition expels them to the bottom and eliminates the effect).

#### Effects of the Detonation Nanodiamonds

##### Pure “end-of-the-cycle” ND

**FIGURE 10:**
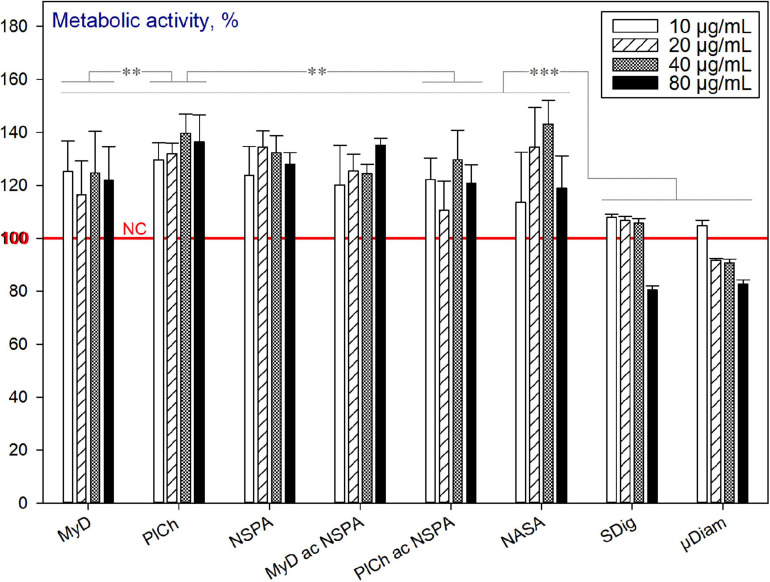
Metabolic activity of HDFs cultured with completely purified (“end-of-cycle”) nano- and micronsized diamonds for 72 h, using the alamarBlue^TM^ assay (normalised to the non-treated cells; *n* = 3; ^∗∗∗^*p* < 0.001; ^∗∗^*p* < 0.01; ^∗^*p* < 0.05; ± SD).

In general, the majority of the studied completely purified ND variants exhibited no cytotoxicity. On the contrary, within the concentration range of 10–80 μg mL^–1^ used, they were well tolerated and an increase in the metabolic activity of cells was observed. Here, under complete purification, we understand the aspect of non-diamond (sp^2^-coordinated) carbon removal. As shown in Figure 10, all of the MW-purified, commercial and even dichromate purified samples increased the metabolic activity of the HDFs in comparison with the non-treated cells (NC: no nanodiamond addition). However, the commercial PL-SDND-02p “single-digit” ND suspension (SDig) at the concentration 80 μg mL^–1^, and HPHT type of micronsized diamond powder (PL-DD-01-0, μDiam) at the concentration of 40 μg mL^–1^ and over, caused a decrease in the metabolic activity. This may be attributed to the influence of the suspension stability additives, as previously revealed (Mitev et al., 2014b).

##### Plasma-assisted Purifications

**FIGURE 11:**
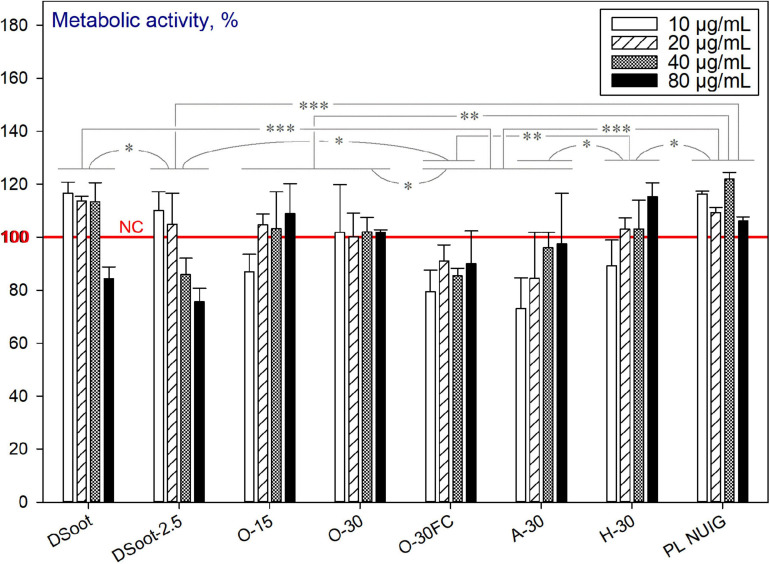
Metabolic activity of HDFs cultured with incompletely sp^2^-C-purified DND, using the alamarBlue^TM^ assay (normalised to the non-treated cells; *n* = 3; ^∗∗∗^*p* < 0.001; ^∗∗^*p* < 0.01; ^∗^*p* < 0.05; ± SD).

The metabolic activity of the HDFs, cultured with the starting material—the diamond soot (DSoot), was initially evaluated, and at concentrations of 10, 20, and 40 μg mL^–1^ showed no cytotoxic effects for the cells (Figure 11). For all other plasma-purification variants, the tolerance of the HDFs to the nanomaterials was significantly reduced compared to DSoot. This correlates to the black and white ratio of the samples, and to large extent, with the sp^2^-carbon presence as revealed by XPS. Moreover, it is worth mentioning that the cytotoxicity of carboxylated NDs was previously described (Roy et al., 2018), as well as the slight reduction of the neuroblastoma cell viability in the presence of carbon black as a control (Schrand et al., 2007).

It can be concluded that the two processes occur concurrently for the partially purified DND (incomplete in the aspect of sp^2^-carbon removal). The first one, as outlined above, is the beneficial effect of the increasing concentration of completely purified (“end-of-cycle”) ND. Secondly, the pronounced cytotoxicity of partially oxidised non-diamond type of carbon, such as sp^2^ graphitic, is very significant here as an opposite effect. To check the last hypothesis, the initial DS was plasma oxygen-treated for just 2.5 min (DSoot-2.5: Figure 11 and Supplementary Information 7). Despite this very short plasma oxidation, the detrimental effect on cell viability was significant. To sum up, with the beginning of the sp^2^-carbon oxidation, the cytotoxicity is induced, which reaches its maximum effects for the incompletely purified “dark” DND samples (O-30FC, A-30). However, by reaching the advanced stages of DND oxidative plasma purification (PL NUIG), the cytocompatibility of the nanoparticles is regained.

##### Microwave-assisted Refinements

**FIGURE 12:**
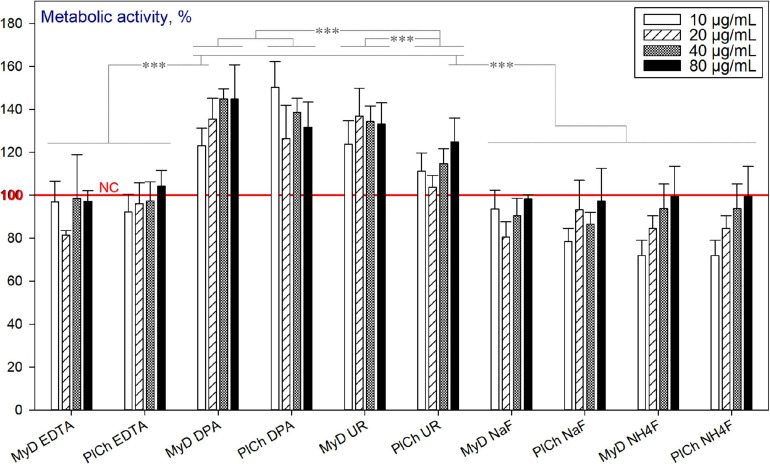
Metabolic activity of HDFs cultured with MW-refined DND samples, using the alamarBlue^TM^ assay (normalised to the non-treated cells; *n* = 3; ^∗∗∗^*p* < 0.001; ^∗∗^*p* < 0.01; ^∗^*p* < 0.05; ± SD).

As discussed previously, the MW-assisted refinement procedures target further purification of DND mainly from the metallic hetero-admixtures and some ‘commonly present’ non-carbon elements, such as Si, Sb, S, and B. However, the sp^2^-carbon is not targeted here but with MW-assisted purifications. Figure 12 depicts the viability of the HDFs in the presence of DND, refined through the protocols outlined before. As can be seen, “DPA”- and “UR”-based refinements output DND with enhanced cytocompatibility, unlike “EDTA” and both fluoride treatments. It should be emphasised that the adverse effects of EDTA have already been observed for murine fibroblast cells (Marins et al., 2012), rat kidney cells (Hugenschmidt et al., 1993), lymphoma and leukaemia-derived cell lines (Galluzzi et al., 2012). Fluorides have been found harmful for murine embryo fibroblasts (Salomão et al., 2017), human mucosal fibroblasts (Jeng et al., 1998), and other cell types. All of the refined DND samples here, such as 0.120–0.122 g of types MyD and PlCh underwent eight consecutive 50 mL in-falcon washings with intermediate centrifugations as a universally established procedure. Apparently, the detrimental effects observed were impinged by EDTA and fluoride ions still adsorbed on NDs, due to their developed surface and good adsorption properties (Peristyy et al., 2014).

##### Plasma-assisted Modifications

**FIGURE 13:**
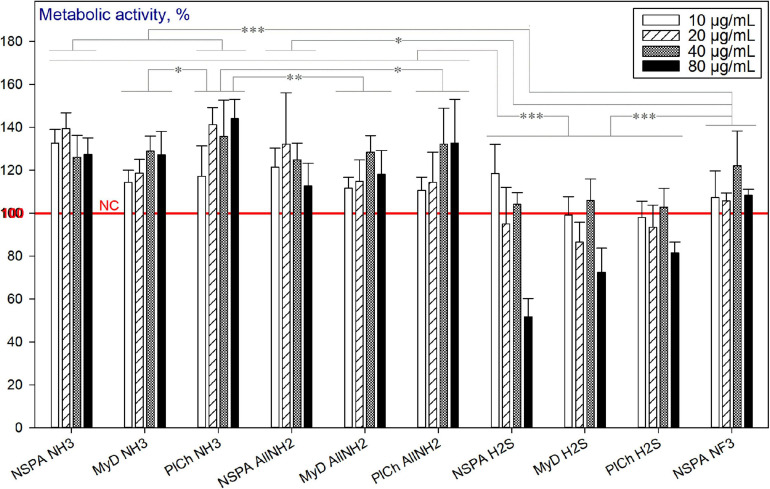
Metabolic activity of HDFs cultured with plasma-modified DND, using the alamarBlue^TM^ assay (normalised to the non-treated cells; *n* = 3; ^∗∗∗^*p* < 0.001; ^∗∗^*p* < 0.01; ^∗^ p < 0.05; ± SD).

Amongst the plasma-modified NDs, only the sulfhydrylation treatment led to reduced viability of HDFs. The effect was pronounced for concentrations of 80 μg mL^–1^ for all three DND types used (Figure 13).

## Conclusion

The purification of detonation soot, based on cold plasma treatment under vacuum is a promising low-cost and green alternative for ND production. Despite being a “dry” method, without the deaggregation option of liquid processes, it altered the ND more radically than the MW alternatives. The application of either the oxidative (O_2_, air) or reductive (H_2_) conditions largely removed the sp^2^-hybridised carbon from the primary product, without a need for liquid-state chemical interventions at that stage. Further microwave-based purification was capable of almost complete elimination of the remaining hetero-elemental admixtures with only the minute use of chemicals. In addition to the useful reduction of certain non-carbon elements, when medical use of the product is pursued, the application of MW-assisted complexant refinement has to be followed by similar oxidative acid treatment for the purpose of removing adsorbed organic and ionic species. The plasma-assisted ND amination, allyl-amination, sulfhydrylation and fluorination were speedy, nature-friendly and convenient procedures for tailoring the nanomaterials’ surface, potentially useful for many further biological and non-biological applications. When the cytotoxicity of DND was concerned, the presence of various non-carbon hetero-elements did not influence the toxicity against HDFs, as revealed by the cell viability assays and optical microscopy tracking. The maintained metabolic activity of HDFs in the presence of these materials was remarkable compared to the untreated cells. This output is even more important as a result of a “dry” approach, without subsequent deaggregation. Enhanced cytotoxicity was only manifested in the presence of DND, only partially purified from sp^2^-carbon admixture through oxidation. Moreover, the established dependence of the ND deposition methods on the cell-related effects is also amongst the merits of this work.

## Data Availability Statement

The original contributions presented in the study are included in the article/Supplementary Material, further inquiries can directed to the corresponding authors.

## Author Contributions

The manuscript was written through contributions of all authors. All authors have approved the final version of the manuscript.

## Conflict of Interest

SW and CD were employed by company Diener Electronic GmbH + Co. KG. The remaining authors declare that the research was conducted in the absence of any commercial or financial relationships that could be construed as a potential conflict of interest.

## Abbreviations

AB: AlamarBlue (cells viability/cytotoxicity assay)
AllNH_2_: allyl-aminated
ATR-FTIR: attenuated total reflection-Fourier-transform infrared spectroscopy
BET: specific surface area by Brunauer-Emmett-Teller (BET) theory
B & W: Black & White (colour ratio)
CA: contact angles
CVD: chemical vapour deposition
DS or DSoot: detonation Soot/Diamond(-containing) Soot
DI: deionised (water)
DND: detonation nanodiamonds
DMEM: Dulbecco’s modified Eagle’s medium
DPA: dipicolinic (2,6-pyridinedicarboxylic) acid
EDTA: (here) disodium salt of EDTA (Na_2_EDTA)
FC (conditions): Faraday Cage (indirect plasma treatment in grid-walled metal box)
FBS: fetal bovine serum
FTIR: Fourier-transform infrared spectroscopy
HPHT: high pressure high temperature type of micronsized diamond powder (static synthesis)
HDFs: human dermal fibroblasts
HAEC: human airway epithelial cells
ICP-MS: inductively coupled plasma-mass spectrometry
μ Diam: micronsized diamond powder (HPHT static synthesis)
MW: microwave (-assisted, -purified etc.)
NC: negative control (cells)
ND: nanodiamond
n: (Suffix “n”): new samples under corresponding abbreviations (Mitev et al., 2014a). Understood here by default, although it may be skipped
NUIG: National University of Ireland, Galway
sccm: Standard cubic centimetres per minute
SEM-EDX: SEM microscope equipped with an EDX detector
PBS: phosphate-buffered saline
PG: PicoGreen (proliferation assay)
PL: plasma-purified plasma-assisted etc.
PTFE: polytetrafluoroethylene (commonly known as Teflon ^TM^)
RF: radio frequency
SDND SDig: deaggregated “single-digit” type of ND
3D: three-dimensional, in three dimensions
UR: hexamethylenetetramine (URotropine)
UV: ultraviolet
XPS: X-ray photoelectron spectroscopy
ZP: zeta potential.
