# Supplementary material for: Plasma & Microwaves as Greener Options for Nanodiamond Purification: Insight Into Cytocompatibility
Source: Front Bioeng Biotechnol. 2021 Jun 30;9:637587. doi: 10.3389/fbioe.2021.637587 (PMC8278578; doi:10.3389/fbioe.2021.637587)
Supplement: Supplementary file 1 [file Data_Sheet_1.docx]

Supplementary Material

**Supporting information 1: Used chemicals and gasses:**

All reagents used for nanodiamond treatment were of analytical and ACS-grade quality. For the microwave-related processing and sample preparation treatments for elemental determination by inductively coupled plasma mass spectrometry (ICP-MS) only suprapure/ultrapure acids and Milli-Q water (18.3 MΩ Milli-Q Element System™, Merck Millipore, USA; pH 5.75) were used. Deionised (DI) water was also used for intermediate washing procedures (16.5 MΩ-cm; pH 5.50); dH_2_O (Ernst Vertriebsgesellschaft mbH) was used for suspensions for plasma sample slides. For the experiments, raw detonation Diamond Soot (DS; DSoot) was obtained from NanoDiamond Products DAC (Ireland; code PM00000UDD00-2UP; country of origin China; BET specific surface 224 m^2^g^-1^); a black powder with a pH of 1% aqueous suspension 6.80. A commercial DND of brand PL-D-G (grade G, lot# YFAK170901) was also acquired from PlasmaChem, Germany, together with de-aggregated ‘single-digit’ SDND suspension and HPHT type of micronsized diamond powder for benchmark purposes (PL-DD-01-0, grade 0.1/0, lot# 1411-5).

Used acids: H_2_SO_4_ min. 95% (p.a.; Fisher Scientific, UK) and 96% (Suprapur; Merck KGaA, Germany); HNO_3_ 67-69% (SpA super pure; Romil™, UK), HClO_4_ 70% (ACS grade; Sigma-Aldrich, USA; now Merck KGaA).

Other chemicals (all Sigma-Aldrich, USA; now Merck KGaA): K_2_Cr_2_O_7_ ≥99.0% (ACS grade); 2,6-Pyridinedicarboxylic acid 99% (DPA); Na_2_EDTA.2H_2_O 98.5-101.5% (Sigma grade); NaF ≥99.0% (ACS grade); NH_4_F ≥98.0% (ACS grade); Hexamethylenetetramine ≥99.0% (UR; ACS grade).

Used gasses: CO_2_ (99.8%; Gase Lüdenbach GmbH, Germany); H_2_S (99.5%; GHC Gerling, Holz + Co., Germany); H_2_ (99.999%; Kraiss & Friz e.K., Germany); O_2_ (100%; Kraiss & Friz e.K., Germany and BOC, Ireland); Ar (99.996%; Kraiss & Friz e.K., Germany and BOC, Ireland); NF_3_ (99.999%; Air Products and Chemicals, Inc., USA); NH_3_ (100%; Kraiss & Friz e.K., Germany).

**Supporting information 2: Instruments and conditions for the plasma (A) and MW (B) procedures**

**(A).** Plasma procedures

All gas precursors were used at 0.250 mbar working pressure (0.280 mbar for O-15). In the case of liquid allyl amine, the vapour pressure under vacuum was high enough to ensure introduction without carrier gas or spraying. Gas flows varied depending on the precursor used. Power modes of 200, 300 and 400 W were used throughout the experiments, with registered temperatures all within the range of 38-47 °C. All purification plasma procedures involved straight application of the corresponding gas (O_2_, H_2_ or air). When bio-modification of already purified nanodiamond was sought, procedures had a prior integrated pre-treatment cleaning step (usually 5-minute Ar bombardment at 60% power; see **Table A**).

**Supplementary Table A.** Procedures applied for plasma purification or surface modification of nanodiamond

| Procedure name | Procedure type | Specifics | Treatment time, *min* | Pre-treatment, time and specifics |
| --- | --- | --- | --- | --- |
| O-15 and O-15FC* | Purification | O_2_; 57 sccm; 400 W | 6 x 15’ | None |
| O-30 and O-30FC | Purification | O_2_; 46 sccm; 200 W | 3 x 30’ | None |
| A-30 and A-30FC | Purification | Air; **; 200 W | 3 x 30’ | None |
| H-30 and H-30FC | Purification | H_2_; 23 sccm; 200 W | 3 x 30’ | None |
| ‘NH_3_’ | Surface modification | NH_3_; 70 sccm; 200 W | 20’ | 5’ (Ar; 35 sccm; 300 W) +  5’ (CO_2_; 19 sccm; 200 W) |
| ‘AllNH_2_’ | Surface modification | Allyl amine; **; 200 W | 5’ | 5’ (Ar; 35 sccm; 300 W) |
| ‘H_2_S’ | Surface modification | H_2_S; 36 sccm; 200 W | 3’ | 5’ (Ar; 35 sccm; 300 W) |
| ‘NF_3_’ | Surface modification | NF_3_; 26 sccm; 300 W | 20’ | None |
| **Suffix FC denotes indirect plasma treatment (Faraday Cage, all grounded, only for O-15FC isolated for the first of the six 15-min cycles)*  *** Manually to the pressure of 0.250 mbar* | | | | |

Plasma systems:

*Tetra 30 LF PC plasma system (Diener electronic GmbH + Co. KG, Ebhausen, Germany).* This capacitive 40 kHz plasma system has an internal working chamber with a volume of 30 L and maximum power load of 500 W. For direct plasma treatments, the nanodiamond/DS sample slides were placed horizontally onto the lower working electrode (a solid stainless steel plate / tray (only O-15 samples) or stainless steel framed grid plate (others). All experiments also comprised a simultaneous indirect downstream/afterglow plasma treatment, where the sample slides were isolated from the charged particles’ bombardment by insertion into a grounded Faraday cage (stainless steel).

**Supplementary Figure 1 S.** Tetra 30 LF PC

**Supplementary Figure 2 S.** Disposition of the samples during the plasma treatment (Tetra)

*Zepto-BLS LF plasma system (Diener electronic GmbH + Co. KG, Ebhausen, Germany).* This laboratory-scale capacitive 40 kHz plasma system has a borosilicate glass working chamber with a volume of 1.5 L, outer electrodes and maximum power load of 30 W. For the direct plasma treatments, the nanodiamond/DS sample slides were placed horizontally onto the bottom of the glass tube chamber, in a crosswise manner. Treatment gasses such as O_2_, air and Ar precursors were only used here, at maximum power modes of 30 W. Gas flow of 60 NL h^-1^ (1000 sccm) was mainly applied throughout the procedures.

**Supplementary Figure 3 S.** Disposition of the test slides during the plasma treatment (Zepto)

**(B).** Microwave procedures

**Supplementary Table B1.** Procedures applied for microwave-assisted purification of nanodiamond

| Name | Components | Ratio,  *vol%* | Volume per vessel,  *mL* | Digested amount  of DS per run,  *g* | Applied average  microwave flux,  *W mL^-1^ sec^-1^* |  |
| --- | --- | --- | --- | --- | --- | --- |
| NSPA (n)* | HNO_3_, H_2_SO_4_, HClO_4_ | 48/46/6 | 5 | 1.1 | 2.288 x 10^-1^ |  |
| NASA (n) | HNO_3_, H_2_SO_4_ | 50/50 | 5 | 1.1 | 2.288 x 10^-1^ |  |
| PANA (n) | HClO_4_, HNO_3_ | 50/50 | 5.5 | 1.1 | 2.080 x 10^-1^ |  |
| ** Suffix ‘n’ denotes new samples under these abbreviations ^19^. Here it is understood by default, although it will be skipped further* | | | | | | |

**Supplementary Table B2.** Procedures applied for microwave-assisted refinement of nanodiamond

| Name  (MyD or PlCh) plus: | Components | Ratio,  *%* | Volume per vessel,  *mL* | DND amount in each vessel,  *g* | Applied average  microwave flux,  *W mL^-1^sec^-1^* |
| --- | --- | --- | --- | --- | --- |
| EDTA | 3.5% Na_2_EDTA in H_2_O | 3.5% | 5.5 | 0.121 | 2.080 x 10^-1^ |
| DPA | 0.55% DPA in H_2_O | 0.55% saturated | 5.5 | 0.121 | 2.080 x 10^-1^ |
| UR | 3.5% UR in H_2_O | 3.5% | 5.5 | 0.121 | 2.080 x 10^-1^ |
| acid NSPA | HNO_3_, H_2_SO_4_, HClO_4_ | 48/46/6 vol. | 5 | 0.121 | 2.288 x 10^-1^ |
| NaF | 3.5% NaF in H_2_O | 3.5% | 5.5 | 0.121 | 2.080 x 10^-1^ |
| NH_4_F | 3.5% NH_4_F in H_2_O | 3.5% | 5.5 | 0.121 | 2.080 x 10^-1^ |
|  | | | | | |

**Supporting Information 3: Elements determined by ICP-MS - normalised to ppm in dry samples**

|  | MyD EDTA | MyD DPA | MyD acid NSPA | MyD NaF | MyD NH4F | PANA | NSPA | MyD (dichrom.; initial) | PlCh (initial) | PlCh EDTA | PlCh DPA | PlCh UR | PlCh acid NSPA | PlCh NaF | PlCh NH4F | O-15 | O-30 | O-30FC | A-30 | H-30 | NASA | DSoot initial |  |
| --- | --- | --- | --- | --- | --- | --- | --- | --- | --- | --- | --- | --- | --- | --- | --- | --- | --- | --- | --- | --- | --- | --- | --- |
| B | 2 | 2 | 2 | 1 | 1 | 3 | 3 | 4 | 69 | 92 | 71 | 33 | 29 | 41 | 64 | 103 | 27 | 24 | 14 | 34 | 0 | 0 | **B** |
| Al | 37 | 64 | 0 | 63 | 6 | 59 | 0 | 64 | 1429 | 1485 | 1547 | 1258 | 18 | 322 | 110 | 249 | 423 | 134 | 345 | 231 | 37 | 159 | **Al** |
| Ti | 4 | 5 | 2 | 2 | 2 | 4 | 2 | 3 | 134 | 250 | 150 | 122 | 62 | 102 | 170 | 10 | 4 | 0 | 4 | 6 | 3 | 3 | **Ti** |
| Cr | 467 | 683 | 188 | 505 | 565 | 6 | 4 | 1929 | 44 | 55 | 34 | 39 | 7 | 25 | 69 | 339 | 211 | 10 | 243 | 195 | 6 | 8 | **Cr** |
| Fe | 0 | 137 | 0 | 94 | 8 | 295 | 279 | 44 | 272 | 34 | 331 | 156 | 27 | 243 | 369 | 2543 | 2786 | 634 | 3007 | 1640 | 314 | 1745 | **Fe** |
| Mn | 0 | 0 | 0 | 0 | 0 | 0 | 0 | 0 | 10 | 3 | 8 | 7 | 2 | 6 | 7 | 41 | 40 | 18 | 25 | 48 | 0 | 10 | **Mn** |
| Ni | 0 | 0 | 0 | 0 | 0 | 0 | 0 | 0 | 4 | 3 | 3 | 3 | 3 | 5 | 5 | 70 | 67 | 3 | 44 | 28 | 0 | 1 | **Ni** |
| Cu | 3 | 3 | 1 | 3 | 3 | 0 | 1 | 2 | 25 | 5 | 15 | 14 | 4 | 20 | 30 | 445 | 395 | 246 | 369 | 356 | 1 | 286 | **Cu** |
| Zn | 2 | 1 | 2 | 2 | 2 | 0 | 1 | 3 | 7 | 0 | 1 | 4 | 88 | 78 | 2 | 138 | 190 | 186 | 178 | 222 | 2 | 229 | **Zn** |
| Ba | 0 | 0 | 0 | 0 | 0 | 0 | 0 | 0 | 0 | 1 | 0 | 0 | 2 | 2 | 1 | 6 | 5 | 3 | 5 | 5 | 0 | 1 | **Ba** |
| Pb | 0 | 0 | 0 | 2 | 2 | 0 | 0 | 2 | 0 | 0 | 0 | 3 | 1 | 0 | 0 | 92 | 90 | 45 | 67 | 80 | 0 | 61 | **Pb** |
| Cd | 0 | 0 | 0 | 0 | 0 | 0 | 0 | 1 | 0 | 0 | 0 | 0 | 0 | 0 | 0 | 846 | 1316 | 981 | 912 | 1455 | 0 | 1324 | **Cd** |
| Sn | 0 | 2 | 3 | 0 | 0 | 8 | 0 | 2 | 21 | 12 | 18 | 12 | 4 | 11 | 13 | 12 | 13 | 13 | 20 | 8 | 9 | 14 | **Sn** |
| Sb | 4 | 10 | 5 | 6 | 5 | 19 | 17 | 9 | 48 | 32 | 51 | 23 | 12 | 20 | 22 | 31 | 28 | 15 | 33 | 25 | 23 | 25 | **Sb** |
| Mo | 1 | 1 | 0 | 1 | 1 | 0 | 0 | 0 | 2 | 2 | 2 | 1 | 2 | 2 | 3 | 8 | 4 | 0 | 5 | 4 | 0 | 0 | **Mo** |
| Bi | 0 | 0 | 0 | 0 | 0 | 0 | 0 | 0 | 32 | 5 | 44 | 15 | 17 | 23 | 41 | 2 | 1 | 1 | 0 | 0 | 0 | 0 | **Bi** |
| W | 1 | 1 | 0 | 0 | 0 | 0 | 0 | 0 | 7 | 9 | 7 | 3 | 3 | 6 | 10 | 1 | 0 | 0 | 0 | 0 | 0 | 0 | **W** |
| Ag | 0 | 0 | 0 | 0 | 0 | 0 | 0 | 0 | 4588 | 3912 | 984 | 3716 | 951 | 1421 | 838 | 0 | 0 | 0 | 0 | 0 | 0 | 0 | **Ag** |

**Supplementary Table C.** Elements determined by ICP-MS - normalised to ppm in dry samples

**Supporting Information 4: XPS elemental composition and ratios of ND samples in at%**

**Supplementary Table D.** Elemental composition and ratios of ND samples in at%; XPS

| Element line |  | Sample (at%; mean ±SD) | | | | |
| --- | --- | --- | --- | --- | --- | --- |
|  | **DSoot** | **O-30** | **O-30FC** | **H-30** | **NSPA** | **NSPA NH_3_** |
| C 1s | 93.96 ±0.11 | 87.50 ±0.44 | 88.51 ±0.23 | 87.02 ±0.52 | 90.61 ±0.25 | 89.08 ±0.70 |
| O 1s | 3.86 ±0.31 | 10.17 ±0.30 | 9.48 ±0.04 | 10.39 ±0.37 | 7.24 ±0.11 | 7.60 ±0.46 |
| N 1s | 1.84 ±0.08 | 1.77 ±0.08 | 2.01 ±0.18 | 2.01 ±0.23 | 1.57 ±0.23 | 2.48 ±0.13 |
| S 2p | 0.22 ±0.11 | - | - | - | 0.13 ±0.08 | 0.10 ±0.04 |
| Na 1s | - | 0.26 ±0.01 | - | 0.36 ±0.00 | - | 0.18 ±0.02 |
| Cl 2p | 0.14 ±0.02 | 0.06 ±0.01 | - | 0.05 ±0.06 | 0.46 ±0.00 | 0.33 ±0.01 |
| F 1s | - | 0.27 ±0.04 | - | - | - | 0.25 ±0.17 |
| Si 2p | - | - | - | 0.19 ±0.01 | - | - |
|  |  |  |  |  |  |  |
| Ratio C: sp^3^/sp^2^ | 34.9/23.9 (59.4%) | 35.2/16.9 (67.6%) | 39.7/11.0  (78.3%) | 40.9/12.3 (76.9 %) | 45.4/7.7 (85.5%) | 46.0/7.3 (86.3%) |
| Elemental bonds distribution | **N:** 1.4 (C-N), 0.4 (N+, NH_4_^+^, Azide), 0.2 (NO_2_^-^);   **C:** 32.8 (C-O, C-N),  1.6 (C=O. O-C=O);   **S:** 0.1 (S^2^**^–^**), 0.2 (sulphone) | **N:** 1.3 (C-N), 0.4 (N+, NH_4_^+^, Azide), 0.1 (NO_2_^-^);  **C:** 32.8 (C-O, C-N),  3.5 (C=O, O-C=O) | **N:** 1.3 (C-N), 0.5 (N+, NH_4_^+^, Azide), 0.2 (NO_2_^-^);  **C:** 35.3 (C-O, C-N),  2.8 (C=O, O-C=O) | **N:** 1.7 (C-N), 0.5 (N+, NH_4_^+^, Azide), 0.1 (NO_2_^-^);  **C:** 32.0 (C-O, C-N),  2.5 (C=O, O-C=O) | **N:** 1.3 (C-N), 0.4 (N+, NH_4_^+^, Azide), 0.1 (NO_2_^-^);  **C:** 35.4 (C-O, C-N),  2.7 (C=O, O-C=O);  **S:** 0.02 (S^2^**^–^**), 0.1 (sulphone) | **N:** 2.0 (C-N), 0.4 (N+, NH_4_^+^, Azide);  **C:** 34.5 (C-O, C-N),  2.4 (C=O, O-C=O);  **S:** 0.1 (sulphone) |

**Supporting Information 5:**

***● Contact angles on thin nanomaterial layers:***

**Supplementary Figure 4 S.** Contact angles on thin nanomaterial layers after different plasma modifications, measurement 3 sec after the drop’s detachment

***● Extremely non-adherent fluorinated nanodiamond:***

**Supplementary Figure 5 S.** Fluorinated layers are so extremely non-adherent and sensitive to static electricity that they slip off the slide upon slightest move

**Supporting Information 6: Proliferation of HDFs cultured with corresponding nanomaterials using the PicoGreen assay - normalized as % vs the non-treated cells**

**Supplementary Table E..**

|  | MyD | PlCh | NSPA | MyD ac NSPA | PlCh ac NSPA | NASA | SDig | µDiam |
| --- | --- | --- | --- | --- | --- | --- | --- | --- |
| 10 µg/mL | 133 | 176 | 78 | 160 | 105 | 119 | 213 | 198 |
| 20 µg/mL | 205 | 194 | 156 | 78 | 88 | 120 | 201 | 148 |
| 40 µg/mL | 158 | 154 | 126 | 85 | 86 | 121 | 472 | 95 |
| 80 µg/mL | 208 | 218 | 191 | 87 | 85 | 117 | 254 | 204 |

|  | DSoot | O-15 | O-30 | O-30FC | A-30 | H-30 | PL NUIG |
| --- | --- | --- | --- | --- | --- | --- | --- |
| 10 µg/mL | 172 | 95 | 129 | 129 | 97 | 108 | 349 |
| 20 µg/mL | 313 | 78 | 109 | 139 | 106 | 150 | 279 |
| 40 µg/mL | 149 | 150 | 120 | 127 | 118 | 156 | 586 |
| 80 µg/mL | 56 | 141 | 124 | 105 | 97 | 113 | 372 |

|  | MyD EDTA | PlCh EDTA | MyD DPA | PlCh DPA | MyD UR | PlCh UR | MyD NaF | PlCh NaF | MyD NH4F | PlCh NH4F |
| --- | --- | --- | --- | --- | --- | --- | --- | --- | --- | --- |
| 10 µg/mL | 41 | 67 | 103 | 95 | 104 | 80 | 67 | 68 | 43 | 70 |
| 20 µg/mL | 47 | 73 | 122 | 76 | 83 | 78 | 44 | 98 | 44 | 50 |
| 40 µg/mL | 50 | 95 | 127 | 97 | 111 | 98 | 43 | 81 | 48 | 101 |
| 80 µg/mL | 39 | 82 | 113 | 83 | 103 | 126 | 49 | 55 | 46 | 73 |

|  | NSPA NH3 | MyD NH3 | PlCh NH3 | NSPA AllNH2 | MyD AllNH2 | PlCh AllNH2 | NSPA H2S | MyD H2S | PlCh H2S | NSPA NF3 |
| --- | --- | --- | --- | --- | --- | --- | --- | --- | --- | --- |
| 10 µg/mL | 158 | 149 | 152 | 140 | 80 | 87 | 143 | 146 | 121 | 163 |
| 20 µg/mL | 229 | 171 | 178 | 131 | 105 | 103 | 111 | 122 | 190 | 206 |
| 40 µg/mL | 201 | 173 | 153 | 118 | 125 | 119 | 96 | 122 | 142 | 190 |
| 80 µg/mL | 167 | 255 | 199 | 95 | 108 | 107 | 53 | 112 | 122 | 237 |

**Supporting Information 7: Supplementary research**

**Supplementary Figure 6 S.** Comparison layout of PL-purified DND (Diener Tetra system): a) O-15 (bottom left), O-15FC (top left) and initial DS (two slides on the right); b) O-30, O-30FC & DS; c) A-30, A-30FC & DS; d) H-30, H-30FC & DS; e) From right (Diener Zepto system): initial DS, after 2.5 min O_2_, after 90 min O_2_ (PL NUIG)
